# Integrative transcriptomic and epigenomic analysis identifies BCL6B as a novel regulator of human pluripotent stem cell to endothelial differentiation

**DOI:** 10.1093/procel/pwaf039

**Published:** 2025-05-03

**Authors:** Yonglin Zhu, Jinyang Liu, Jia Wang, Shuangyuan Ding, Hui Qiu, Xia Chen, Jianying Guo, Peiliang Wang, Xingwu Zhang, Fengzhi Zhang, Rujin Huang, Fuyu Duan, Lin Wang, Jie Na

**Affiliations:** Center for Regeneration, Aging and Chronic Diseases, School of Basic Medical Sciences, Tsinghua University, Beijing 100084, China; Center for Regeneration, Aging and Chronic Diseases, School of Basic Medical Sciences, Tsinghua University, Beijing 100084, China; SXMU–Tsinghua Collaborative Center for Frontier Medicine, Shanxi Medical University, Taiyuan 030001, China; Center for Regeneration, Aging and Chronic Diseases, School of Basic Medical Sciences, Tsinghua University, Beijing 100084, China; Center for Regeneration, Aging and Chronic Diseases, School of Basic Medical Sciences, Tsinghua University, Beijing 100084, China; School of Life Sciences, Tsinghua University, Beijing 100084, China; Center for Regeneration, Aging and Chronic Diseases, School of Basic Medical Sciences, Tsinghua University, Beijing 100084, China; Center for Regeneration, Aging and Chronic Diseases, School of Basic Medical Sciences, Tsinghua University, Beijing 100084, China; Center for Reproductive Medicine, Department of Obstetrics and Gynaecology, Peking University Third Hospital, Beijing 100191, China; Center for Regeneration, Aging and Chronic Diseases, School of Basic Medical Sciences, Tsinghua University, Beijing 100084, China; Center for Regeneration, Aging and Chronic Diseases, School of Basic Medical Sciences, Tsinghua University, Beijing 100084, China; Center for Regeneration, Aging and Chronic Diseases, School of Basic Medical Sciences, Tsinghua University, Beijing 100084, China; Central laboratory, The First Hospital of Tsinghua University, Beijing 100016, China; Center for Regeneration, Aging and Chronic Diseases, School of Basic Medical Sciences, Tsinghua University, Beijing 100084, China; School of Life Sciences, Tsinghua University, Beijing 100084, China; CAS Key Laboratory of Regenerative Biology, Guangzhou Institutes of Biomedicine and Health, Chinese Academy of Sciences, Guangzhou 510530, China; Center for Regeneration, Aging and Chronic Diseases, School of Basic Medical Sciences, Tsinghua University, Beijing 100084, China; Faculty of Synthetic Biology, Shenzhen University of Advanced Technology, Shenzhen 518017, China; Center for Regeneration, Aging and Chronic Diseases, School of Basic Medical Sciences, Tsinghua University, Beijing 100084, China; State Key Laboratory of Genetic Resources and Evolution, Kunming Institute of Zoology, Chinese Academy of Sciences, Kunming 650223, China; Center for Regeneration, Aging and Chronic Diseases, School of Basic Medical Sciences, Tsinghua University, Beijing 100084, China; SXMU–Tsinghua Collaborative Center for Frontier Medicine, Shanxi Medical University, Taiyuan 030001, China; State Key Laboratory for Complex, Severe and Rare Diseases, Tsinghua University, Beijing 100084, China

**Dear Editor,**

Due to the inaccessibility of early human embryos, little is known about the chromatin status during early human endothelial cell (EC) development. Despite studies showing the epigenomic landscape of primary EC lines or human pluripotent stem cell (hPSC)-derived ECs, the epigenetic dynamic and feature of intermediate progenitors, such as vascular mesoderm cells (VMCs) and endothelial progenitor cells (EPCs), are less known. Therefore, an epigenomic roadmap of human EC development may provide new knowledge about nascent EC formation.

The dynamic change of the epigenetic landscape sheds light on the gene regulatory hierarchy of human EC *de novo* formation. During development, the chromatin regions of key cell fate regulators often open up prior to gene expression. The distribution patterns of active and repressive histone marks closely correlate with cell type and state. Moreover, important *cis-*regulatory elements (CREs), such as enhancers and promoters, are located in open chromatin regions and marked by active histone modifications. ATAC-seq and ChIP-seq have been widely used for epigenetic studies. ATAC-seq reveals the open chromatin, while histone modifications captured by ChIP-seq enable prompt gene transcriptional regulation. For example, trimethylation of histone H3 at lysine 4 (H3K4me3) and lysine 27 (H3K27me3) are considered markers for actively transcribed and silenced genes, respectively. Whereas acetylation at lysine 27 (H3K27ac) is considered a maker for enhancers (Atlasi and Stunnenberg, 2017). Enhancers are cell type and stage-specific and crucial for the spatiotemporally controlled gene expression during embryo development (Long et al., 2016). Therefore, the genomic regions with accessible chromatin and histone modifications, such as H3K4me3, H3K27ac, and H3K27me3, could be used to identify developmentally important CREs for cell fate determination. Here, we systematically depicted the epigenomic landscape of human EC formation using a stepwise differentiation system. The open chromatin, H3K4me3 broad domain, and CRE catalogs provided a comprehensive annotation of the epigenetic roadmap for EC formation from hPSCs. This information also revealed an endothelial-specific transcription factor (TF), BCL6B, which regulates arterial or venous gene networks and EC behavior through Notch signaling.

We used a previously established protocol to obtain key intermediate progenitor cells for EC differentiation (Zhang et al., 2021). VMCs were induced using a combination of BMP4 and CHIR99021 (a GSK3 inhibitor and activator of canonical WNT signaling) for 3 days from human embryonic stem cells (hESCs). And then continued for EC induction for 5 days (Figs. 1A and S1A). On Day 8, 48.1% cells expressed typical EC markers CD31 and CD144 (Fig. S1B and S1C). These cells could take up acetylated low-density lipoprotein (Ac-LDL) and form tubular-like networks on Matrigel (Fig. S1D and S1E), suggesting that they are functional ECs. As early as Day 5, CD31^+^ cells began to appear, alongside the highest FLK1^+^ percentage and transient high *ETV2* expression in FLK1^+^CD31^−^ cells (Fig. S1F–H), indicating Day 5 could be a key transitional point during EC formation. Therefore, we named Day 5 FLK1^+^CD31^−^ and FLK1^+^CD31^+^ cells as EPC-1 and EPC-2, respectively. To acquire the open chromatin landscape of progenitor subpopulations, we sorted Day 3 FLK1^+^ VMC, Day 5 EPC-1, EPC-2, and Day 8 CD31^+^CD144^+^ EC and generated paired ATAC-seq and RNA-seq libraries (Fig. 1A). Principal component analysis (PCA) of ATAC-seq accessible peaks showed a continuous trajectory from hESC to EC (Fig. 1B). Accessible chromatin status and gene expression of key developmental marker genes (*GATA4*, *TAL1*, and *FLK1*) exhibited a strong positive correlation (Fig. 1C). For example, cardiovascular mesoderm marker *GATA4* was open and highly expressed in VMCs, but *TAL1* promoter region was not accessible until EPC stage. We identified six clusters of differentially accessible peaks using k-means clustering. Cluster C5 contained 1,165 peaks which were highly enriched in EPCs and ECs (Fig. 1D). Accordingly, genes with minimal proximity to C5 peaks were relatively highly expressed in EPCs and ECs (Fig. 1E) and were predominantly involved in endothelium development (Fig. 1F). Besides, C5 open chromatin regions were abundant in motifs for developmentally important endothelial ETS family TFs, such as ERG, ETV2, and ETS1 (Fig. 1G). Interestingly, the transcript of *ETV2* was first expressed in EPCs and then followed by *ETS1* and *ERG* expression at a later stage (Fig. 1H). These results obviously reflected the ETS switching mechanism during EC development.

**Figure 1.**
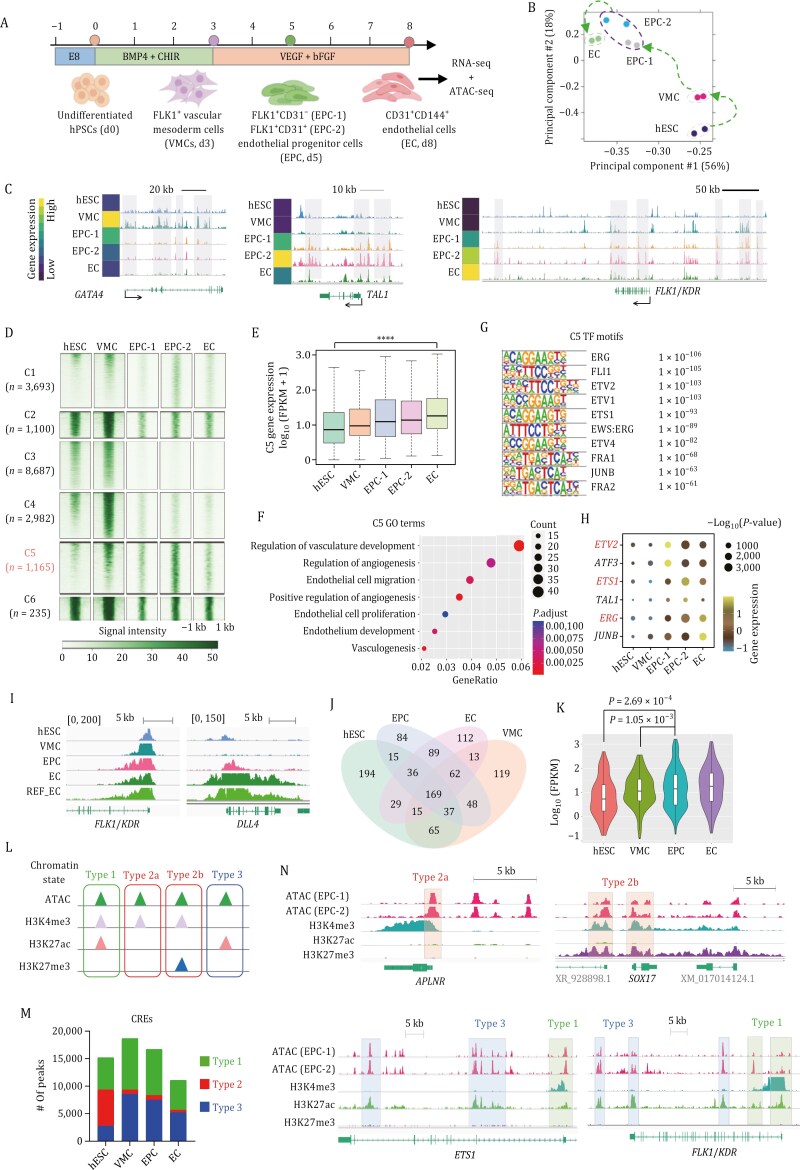
Epigenomic landscape of EC differentiation. (A) Schematic representation of cell purification and sample collection during EC differentiation, outlining the workflow for ATAC-seq and RNA-seq library preparation. CHIR: CHIR99021. (B) PCA of ATAC-seq samples based on differential binding affinity score (re-clustered peak read counts generated using DiffBind). (C) Representative track view of marker genes with expression scale (normalized gene counts from RNA-seq). (D) Heatmap of differential open chromatin peaks of the six clusters using k-means clustering. Signal intensity is calculated by counting the number of reads within 200 bp upstream and downstream of each peak center. (E) Normalized mRNA expression levels of ATAC-seq Cluster 5 (C5) peak-associated genes. Statistical significance was analyzed with one-way ANOVA. ****P < 0.0001. (F) Bubble plot showing C5 peak-associated genes enriched gene ontology (GO) terms. (G) Cluster 5 (C5) open chromatin region enriched TF motifs. (H) Bubble plot showing representative TF motifs identified from open chromatin regions and the corresponding normalized gene expression from RNA-seq (*z*-score normalization). (I) Representative Integrative Genomics Viewer (IGV) snapshot of genes with broad H3K4me3 domain. Scale bars: 5 kb. HUVEC are used as a primary EC reference (Data from GSE53998). (J) Venn diagram showing the number of overlapped broad peak-associated (BPA) genes in hESC, VMC, EPC, and EC. (K) Violin plot showing Day 5 BPA gene expression in hESC, VMC, EPC, and EC. (L) Schematic of CREs classification based on the chromatin state. (M) Numbers of the three types of CRE at each stage of EC differentiation. (N) Representative IGV snapshot of each type of CRE in EPCs.

Next, we profiled the genome-wide binding of the key histone marks and compared them with the open chromatin. Broad H3K4me3 domains are linked with increased elongation, paused polymerase, and enhanced transcriptional consistency (Benayoun et al., 2014). Notably, the top 5% of the broadest H3K4me3 domains preferentially mark cell identity genes. In both ECs and human umbilical vein endothelial cells (HUVECs), the promoter region of *FLK1* and *DLL4* were covered by broad H3K4me3 peaks (>5 kb) (Fig. 1I). Genes marked by broad peaks and specifically enriched in EPCs had significantly higher expression levels compared with their expression in hESCs and VMCs (Fig. 1J and 1K). Furthermore, we integrated open chromatin and histone modification datasets to classify three types of CREs based on their chromatin features. Type 1 CRE was characterized by the co-occurrence of accessible chromatin, H3K4me3, and H3K27ac signals. Type 2 and type 3 CREs exhibited open chromatin peaks overlapping with either H3K4me3 or H3K27ac signals. The type 2 CREs could be further classified into two subtypes: H3K4me3 only (type 2a) and H3K4me3-H3K27me3 dual modification (type 2b) (Fig. 1L). In differentiated cells, Type 1 and Type 3 CREs accounted for the majority of all CREs, whereas type 2 CREs were more prevalent in undifferentiated hESCs (Fig. 1M). For example, in EPCs, the promoter region of *APLNR* and *SOX17* contained type 2a and type 2b CREs, while those of *FLK1* and *ETS1* harbored type 1 CREs (Fig. 1N), suggesting that these CREs may have different functions and epigenetic regulatory mechanisms. Besides, CRE-associated genes were highly related to cell fate specification (Fig. S2). Sum above, our integrative analysis provided rich information about the dynamic change of important epigenetic features during EC differentiation.

To uncover potential new regulators for EC differentiation, we first profiled stage-specific TFs from RNA-seq data. The well-known TFs for endothelial differentiation, such as *ETV2* and *HEY1*, were enriched in EPCs or ECs. We identified 58 TFs specifically expressed in EPCs or ECs. To narrow down the candidates, we combined two published hPSC-EC differentiation scRNA-seq datasets and identified 8 TFs (*BCL6B*, *HOPX*, *LYL1*, *MECOM*, *NFIB*, *SOX18*, *SOX6*, and *TAL1*) with restricted expression in endothelial lineages from both single-cell and bulk RNA-seq data (Figs. 2B, S3A and S3B). Among the candidate TFs, HOPX, NFIB, and SOX6 do not exclusively function in vascular development (Hagiwara, 2011; Palpant et al., 2017; Steele-Perkins et al., 2005), while TAL1, MECOM, SOX18, and LYL1 have been extensively studied or have redundant TFs (Kamachi and Kondoh, 2013; Lv et al., 2023; Pinet et al., 2014). Interestingly, *BCL6B*, but not its paralog *BCL6*, is specifically expressed in ECs (Fig. S3B). Moreover, in mouse studies, BCL6B has been shown to regulate skin angiogenesis and neovascularization of the eye (Ohnuki et al., 2012; Tanaka et al., 2023). Therefore, we decided to choose BCL6B for further validation. In EPCs, the promoter region of *BCL6B* became accessible and was marked by active histone marks, H3K4me3 and H3K27ac (Fig. 2C). *BCL6B* mRNA was highly expressed in CD31^+^ EPCs and ECs (Fig. S4B). BCL6B protein was exclusively in ECs but not in perivascular or stromal cells (Figs. 2D, 2E, and S4A). Next, to find out the role of BCL6B in EC differentiation, we knocked out *BCL6B* and picked two mutant clones, KO-1 and KO-2, for further analysis (Figs. 2F–G and S5A–D). BCL6B KO cells showed enhanced EC differentiation, as indicated by a slightly increased CD31^+^ cell population (Fig. 2H and 2I), but weakened tube formation ability (Fig. S5E and S5F). To evaluate the transcriptional changes resulting from BCL6B KO, we sorted wild-type (WT) and BCL6B KO ECs and performed RNA-seq. Compared with WT ECs, genes upregulated in BCL6B KO ECs were enriched in the Notch signaling pathway and arterial EC differentiation (Fig. 2J and S5G-H), indicating a potential role for BCL6B in arteriovenous specification. Reciprocally, we also generated an inducible BCL6B over-expression (OE) H1 line and performed EC differentiation (Figs. 2K, 2L, and S6A). As expected, RNA-seq analysis showed that doxycycline (dox)-induced BCL6B OE ECs significantly down-regulated arterial EC (AEC) and Notch signaling genes (*DLL4*, *CXCR4*, *EFNB2*, *JAG2*, *NOTCH1*,*2*, etc.), while marked elevated venous EC (VEC) genes (*LYVE1*, *NT5E*, *NR2F2*, etc.) (Figs. 2M and S6B–D). The proportion of CD184^+^CD73^+^ AECs in the BCL6B KO group exceeded 50%, compared with 26.6% in the WT group (Fig. 2N). Conversely, BCL6B OE reduced CD184^+^CD73^+^ AEC generation (48.8% in −dox vs. 15.3% in +dox) but augmented CD184^−^CD73^+^ VEC phenotype (29.0% in −dox vs. 71.6% in +dox) (Fig. 2O). Collectively, the above results suggested that BCL6B acts as a negative regulator of arterial EC gene network.

**Figure 2.**
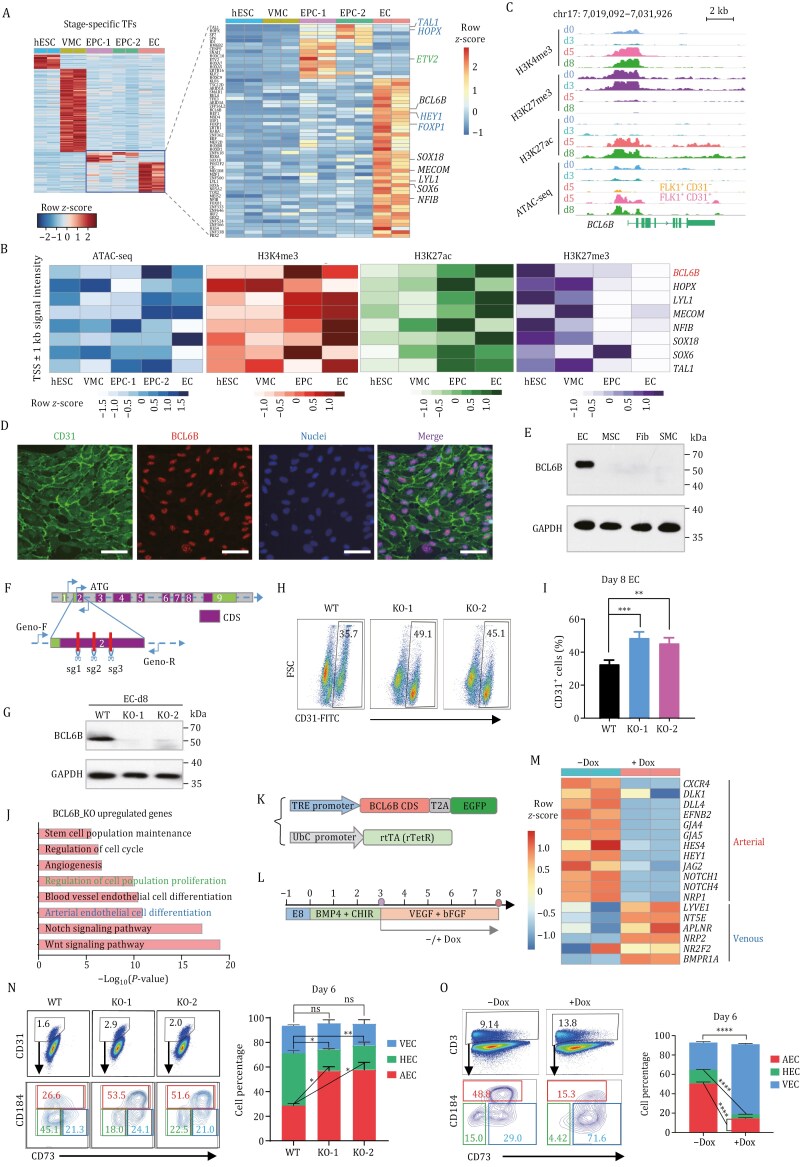
Identify and validate BCL6B as a regulator of EC differentiation. (A) Heatmap of stage-specific TFs expression during EC differentiation. The color scale showed the row *z*-score of gene expression (FPKM value). (B) Epigenetic mark signal intensity of candidate TFs near transcript start site (TSS) region. The color scale shows row *z*-score of RPKM value within TSS ± 1 kb region. (C) IGV view of histone modification (H3K4me3, H3K27me3, and H3K27ac) and ATAC-seq signal enrichment around *BCL6B* promoter region during EC differentiation. (D) Co-staining of BCL6B and CD31 in Day 8 ECs. Scale bars: 100 μm. (E) Western blot showing exclusive BCL6B protein expression in ECs but not in perivascular or stromal cells, including umbilical cord mesenchymal stem cells (MSC), human skin fibroblasts (Fib) and hPSC-derived smooth muscle cells (SMCs). (F) Schematic of sgRNA target sites at *BCL6B* exon 2 to induce gene knockout. (G) Western blot of BCL6B knockout validation. (H and I) Flow cytometry analysis and quantification of CD31^+^ cells in WT and BCL6B^−/−^ cells. Data are presented as mean ± SEM. *n* = 3 biological replicates. **P < 0.01, ***P < 0.001. (J) GO enrichment analysis of significantly upregulated genes in Day 8 BCL6B^−/−^ ECs compared with WT ECs based on bulk RNA-seq. (K) Illustration of BCL6B inducible expression constructs. (L) Schematics of Dox induction of BCL6B expression and EC differentiation. (M) Heatmap showing arterial and venous EC marker gene expression in Day 8 ECs without (−dox) and with (+dox) BCL6B OE based on bulk RNA-seq. The color scale showed row *z*-score of gene expression (FPKM value). (N) Flow cytometry analysis and quantification of arterial (AEC, CD31^+^CD184^+^CD73^−/+^), venous (VEC, CD31^+^CD184^−^CD73^+^), and hemogenic (HEC, CD31^+^CD184^−^CD73^−^) ECs differentiated from WT and BCL6B^−/−^ cells (d6). Data are presented as mean ± SEM. *n* = 3 biological replicates. *P < 0.05, **P < 0.01. (O) Flow cytometry analysis and quantification of AEC, VEC, and HEC percentage after induced BCL6B OE (+dox) vs. control cells (−dox). Data are presented as mean ± SEM. *n* = 3 biological replicates. ****P < 0.0001.

BCL6B, a ZBTB TF, functions as a transcriptional repressor and plays a critical role in mouse retinal vascular development, wound healing-associated angiogenesis, and ocular vascular diseases by downregulating Notch signaling (Ohnuki et al., 2012; Tanaka et al., 2023). Consistent with these reports, we observed BCL6B KO or OE perturbed Notch signaling during hPSC-EC differentiation (Figs. 2J and S6B). Given Notch signaling’s role in arterial EC development, we propose that BCL6B modulates its activity to influence arteriovenous fate. Previous studies demonstrated that *Dll4* haploinsufficiency in mice causes arterial defects (Duarte et al., 2004), whereas *Notch4* over-expression induces arteriovenous malformations (Carlson et al., 2005), underscoring the importance of Notch signaling intensity in EC specification. Additionally, BCL6B was recently shown to repress ETV2, an essential EC fate regulator (Li et al., 2024). We observed an inverse expression pattern between BCL6B and ETV2 during EC differentiation, with *BCL6B* upregulation coinciding with *ETV2* decline in Day 5 EPCs (Figs. S1H and S4B). Both studies demonstrated increased EC generation upon BCL6B depletion, suggesting that BCL6B might be a gatekeeper of EC fate commitment.

Despite advances in hPSC-based cell models, current monolayer differentiation protocols struggle to generate mature arterial or venous subtypes and lack the tissue microenvironment. Recent advances and our study will enhance our understanding of arteriovenous diversification from hPSCs and facilitate future applications in disease modeling and drug screening to identify potential therapeutics.

In conclusion, through integrative multi-omics analyses, we identify BCL6B as a key regulator of arteriovenous specification via Notch signaling, providing a framework for dissecting the transcriptional and epigenetic regulation from pluripotency to endothelial differentiation.

## Supplementary Material

pwaf039_Supplementary_Figures_S1-S5

## Footnotes

This work was supported by the National Key R&D Program of China Grants 2022YFA1103103 and 2023YFA1800302 and the National Natural Science Foundation of China (NSFC) Grants 32270784 and 31970819 to J.N.

Yonglin Zhu, Jinyang Liu, Jia Wang, Shuangyuan Ding, Hui Qiu, Xia Chen, Jianying Guo, Peiliang Wang, Xingwu Zhang, Fengzhi Zhang, Rujin Huang, Fuyu Duan, Lin Wang, and Jie Na declare that they have no conflict of interest. This article does not contain any studies with human or animal subjects performed by any of the authors. All authors give consent to the participation and publication of this work.

Raw and processed RNA-seq, ATAC-seq, and ChIP-seq data are publicly available at the Gene Expression Omnibus with accession number GSE186755. All other relevant data are available from the corresponding author upon request.

Y.Z. and J.N. conceived and designed the experiments. Y.Z. performed cell differentiation, cell line generation, ATAC-seq, ChIP-seq, and RNA-seq library preparation, and bioinformatics analysis. J.L., J.W., S.D., H.Q., X.C., J.G., P.W., F.Z., R.H., F.D., and L.W. participated in data collection, analysis, and assembly. Y.Z. and J.N. wrote the manuscript. All authors approved the manuscript.
