# Supplementary material for: Integrative transcriptomic and epigenomic analysis identifies BCL6B as a novel regulator of human pluripotent stem cell to endothelial differentiation
Source: Protein Cell. 2025 May 3;16(11):985–90. doi: 10.1093/procel/pwaf039 (PMC12698184; doi:10.1093/procel/pwaf039)
Supplement: pwaf039_Supplementary_Figures_S1-S5 [file pwaf039_supplementary_figures_s1-s5.pdf]

Supplementary figures for

Integrative transcriptomic and epigenomic analysis identifies BCL6B as a novel regulator of human pluripotent stem cell to endothelial differentiation

Yonglin Zhu, Jinyang Liu, Jia Wang, Shuangyuan Ding, Hui Qiu, Xia Chen, Jianying Guo, Peiliang Wang, Xingwu Zhang, Fengzhi Zhang, Rujin Huang, Fuyu Duan, Lin Wang, and Jie Na

This file contains

Supplementary figure S1-S6

Supplementary Figures

Fig. S1

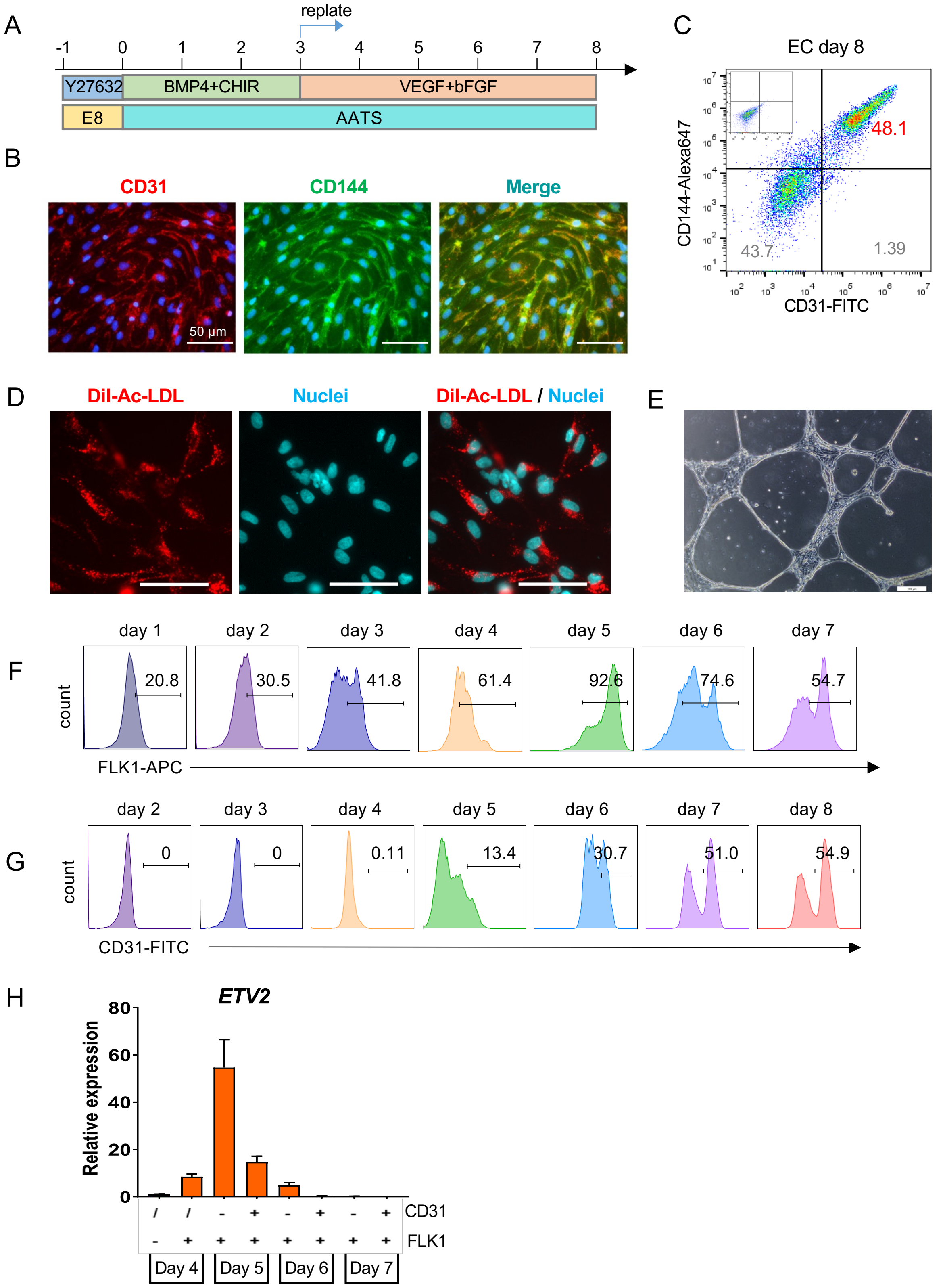

Fig. S1 Characterization of ECs derived from hPSCs. Related to Figure 1.
(A) Schematics of hPSC-EC differentiation. **(B)** Immunostaining of CD31 (red) and CD144 (green) in ECs (H1 derived, d8). Scale bars: 50 μm. **(C)** Flow cytometry analysis of ECs co-stained with CD31 and CD144. **(D-E)** EC *in vitro* functional assays: Ac-LDL (red) uptake **(D)** and tube formation **(E)**. **(F-G)** Flow cytometry analysis of FLK1 and CD31 during EC differentiation. **(H)** Q-PCR of *ETV2* expression in FLK1$^+$, FLK1$^+$CD31$^-$, and FLK1$^+$CD31$^+$ cells during EC differentiation. Data are presented as mean ± SEM. n = 3 biological replicates. **p<0.01, ***p<0.001.

Fig. S2

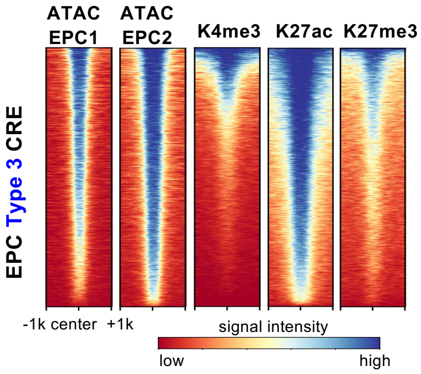

GO terms (BP)	-log10 (p-value)
morphogenesis of a branching structure	8.79
epithelial tube morphogenesis	8.50
vascular process in circulatory system	7.33
endothelium development	6.25
endothelial cell migration	6.04
stem cell population maintenance	5.26
regulation of vasculature development	5.16
ephrin receptor signaling pathway	5.06
artery development	4.54
regulation of angiogenesis	4.00
vasculogenesis	3.94

KEGG pathways	-log10 (p-value)
Rap1 signaling pathway	10.86
Apelin signaling pathway	6.68
cAMP signaling pathway	5.80
Hippo signaling pathway	4.88
Sphingolipid signaling pathway	4.57
MAPK signaling pathway	3.88
VEGF signaling pathway	2.62

Fig. S2 CRE identification and annotation. Related to Figure 1.
Heatmap of type 3 CRE in EPC and associated GO term and KEGG pathway analysis. Note that the enriched GO terms include endothelium development, while KEGG pathway analysis highlights the Apelin signaling pathway, both of which are closely associated with EPC fate specification.

Fig. S3

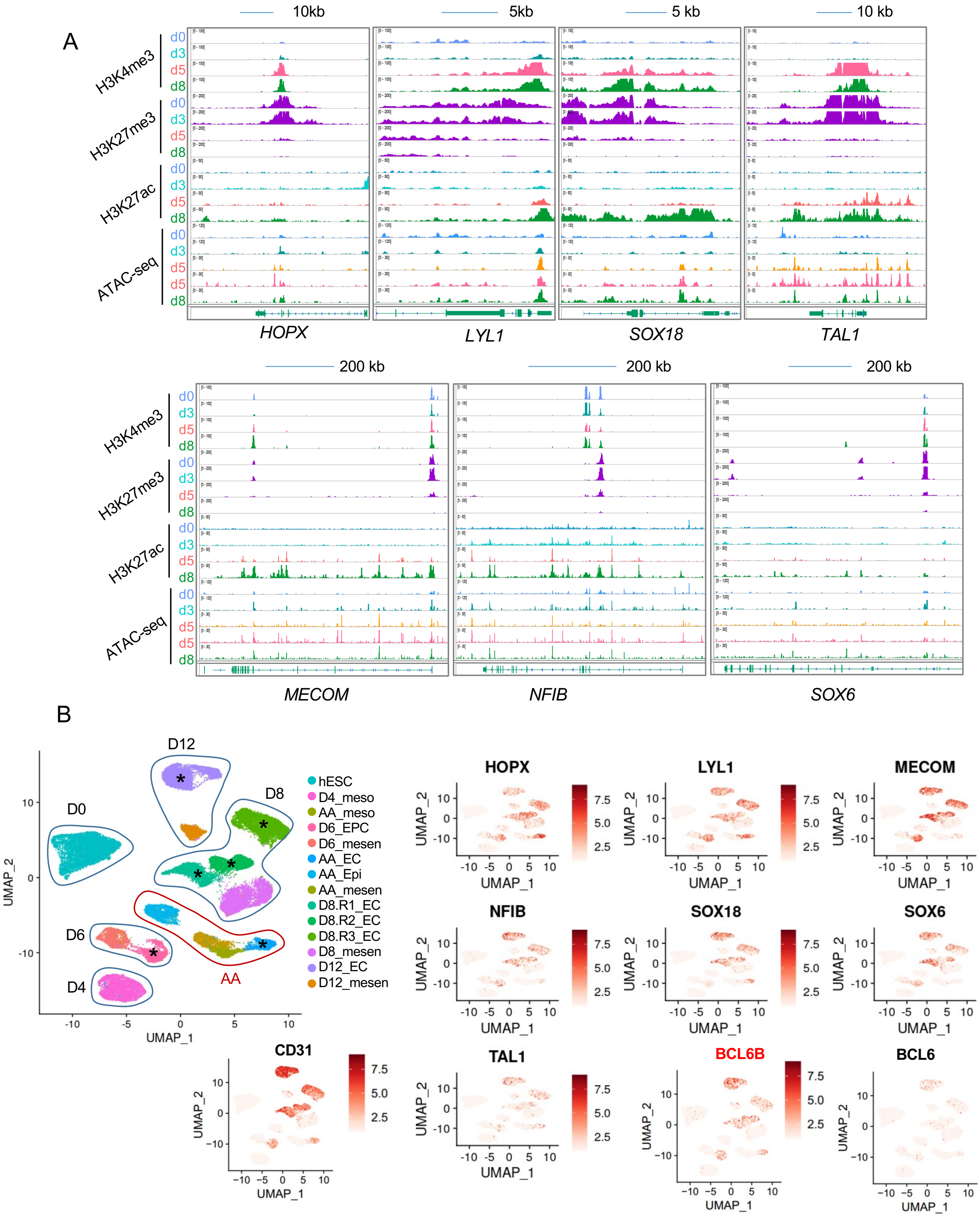

Fig. S3 Identify candidate TFs specifically expressed in endothelial lineages. Related to Figure 2. **(A)** IGV snapshot of histone modification and open chromatin profile for candidate TFs. **(B)** UMAP plot showing co-expression of candidate with CD31 and FLK1 during EC differentiation. AA stands for sample from Zhang's protocol (GSE166462), the others are from McCracken's protocol (GSE131736). Epi, Epithelial cells; mesen, mesenchymal cells; meso, mesoderm cells. Asterisk (*) labels all the EC clusters.

Fig. S4

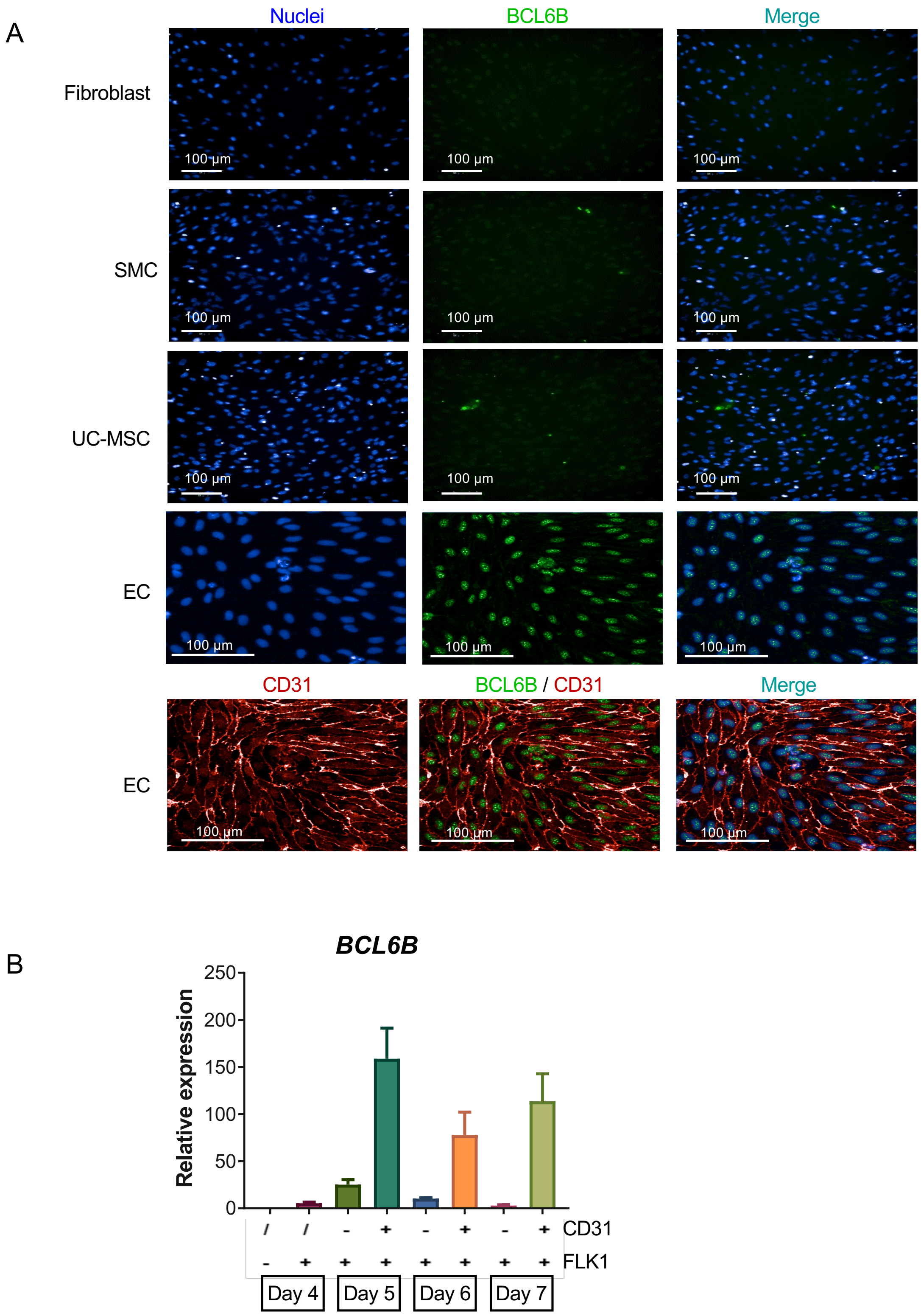

Fig. S4 BCL6B is specifically expressed in human endothelial cells. Related to Figure 2.
(A) Immunofluorescence detecting BCL6B protein in ECs, umbilical cord MSC (UC-MSC), human skin fibroblasts (Fib), and SMCs (derived from H1 hESC). Note that BCL6B (green) is only detected in ECs. (**B**) Q-PCR of *BCL6B* mRNA levels in different progenitor population (FLK1+, FLK1+CD31-, and FLK1+CD31+) during EC differentiation. Data are presented as mean ± SEM. n = 3 biological replicates. $^{**}p<0.01$, $^{***}p<0.001$.

Fig. S5

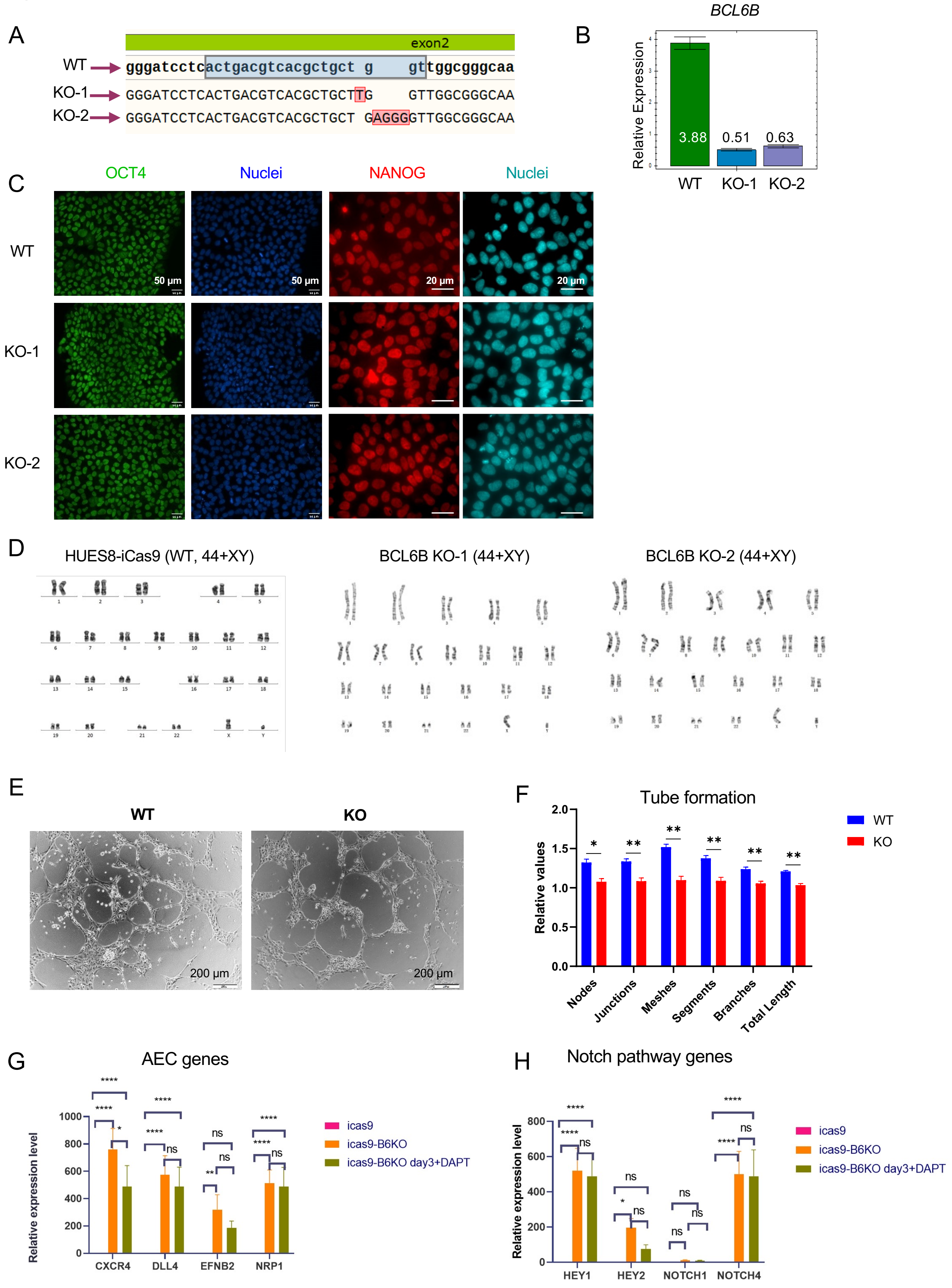

Fig. S5 BCL6B functional studies during hPSC-EC differentiation. Related to Figure 2.
(A-B) Sanger sequencing **(A)** and RT-qPCR **(B)** verification of BCL6B mutant iCas9-HUES8 lines. **(C)** Immunostaining of OCT4 (green) and NANOG (red) in wild-type (WT, iCas9) and BCL6B mutant lines. **(D)** Normal karyotype of WT and BCL6B mutant lines. **(E)** Representative images of tube formation from WT and BCL6B KO ECs. **(F)** Quantification of tube formation using ImageJ Angiogenesis Analyzer. *p<0.05, **p<0.01, p<0.0001****. **(G, H)** Q-PCR analysis showing increased expression of AEC marker genes (G) and selective Notch pathway genes (H) expression in icas9-B6KO. Data are presented as mean ± SEM. n = 3 biological replicates. **p<0.01, ***p<0.001. Note that DAPT, a γ-secretase inhibitor, did not abolish the up-regulation of AEC and Notch pathway genes, suggesting BCL6B regulates these genes at transcriptional level.

Fig. S6

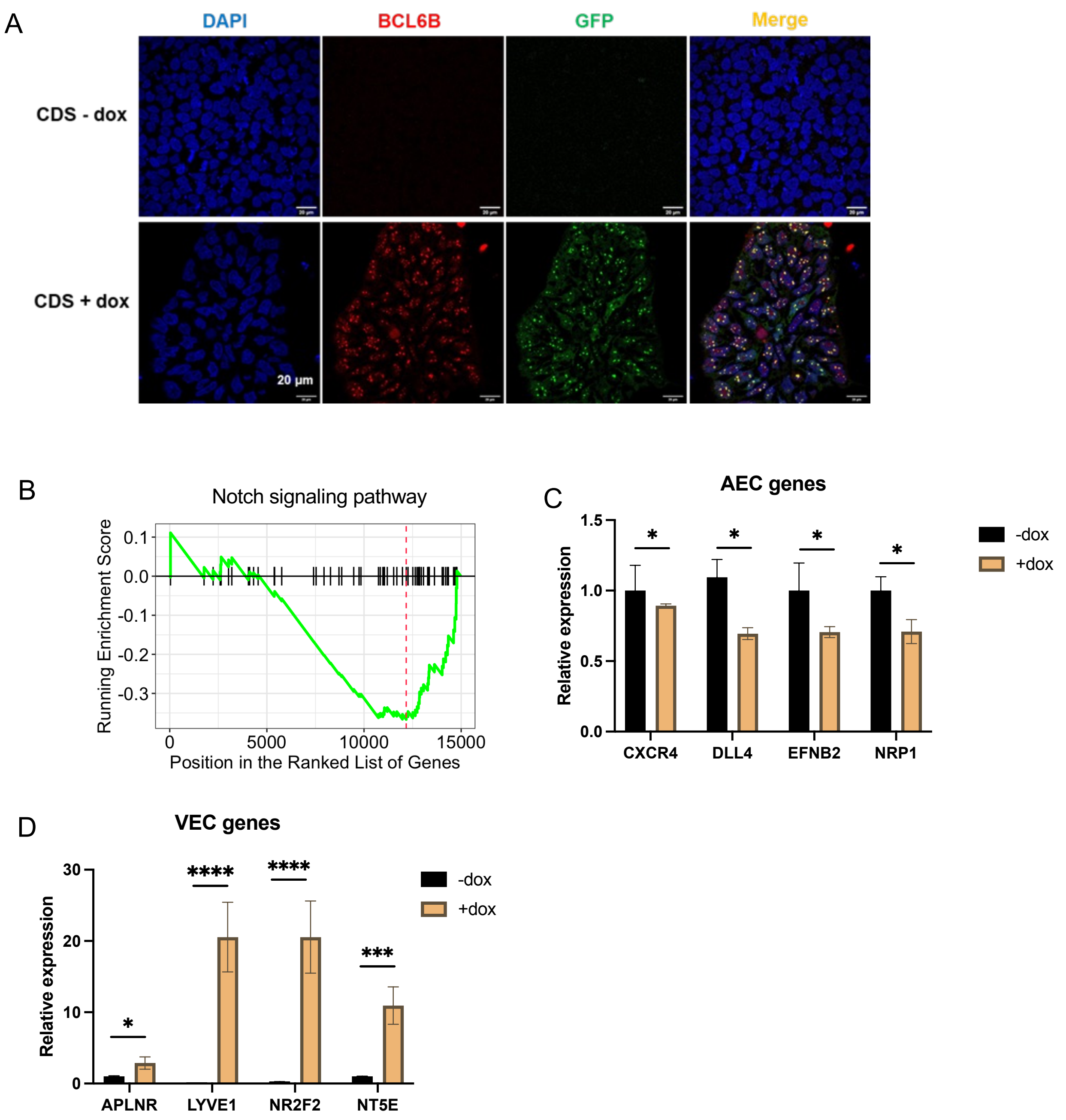

Fig. S6 BCL6B overexpression upregulated VEC genes. Related to Figure 2.
(A) BCL6B (red) and GFP are co-expressed in cell nuclei after doxycycline (CDS+dox) treatment. Scale bars, 20µm. **(B)** Gene set enrichment analysis (GSEA) of differential genes from BCL6B OE (+dox) EC compared with control EC (-dox). **(C, D)** Q-PCR showing the differential arterial and venous EC gene expression in BCL6B OE (+dox) EC and control EC (-dox). Data are presented as mean ± SEM. n = 3 biological replicates. **p<0.01, ***p<0.001.

Materials and Methods

Human pluripotent stem cell culture and differentiation

H1 hESCs (WiCell Institute), HUES8-iCas9 hESCs were routinely maintained on inactivated MEF feeders in the hESC medium, as described previously (Zhang et al., 2017). For feeder-free culture, hPSCs were cultured on vitronectin or Matrigel (BD Biosciences, 356231)-coated plates in TeSR-E8 medium (STEMCELL Technologies, 05990). Cells were dissociated with Accutase (Millipore, SF006) for passaging. The AATS medium for EC differentiation was described in Zhang et al. (Zhang et al., 2021). Briefly, for vascular mesoderm induction, 5 ng/mL BMP4 (Peprotech, 120-05) and 2 μM CHIR99021 (Tocris, 4423) were added to the AATS medium for 2 days with daily medium change. Cells were then splitted and replated onto 50 ng/mL VEGF-A (SinoBiological, 11066-HNAB) and 10 ng/mL bFGF/FGF2 (SinoBiological, 10014-HNAE) supplemented AATS medium for EC induction for another 5 days with daily medium change.

Immunostaining, flow cytometry analysis and Western blot

Primary antibodies used in this study are: (1) immunofluorescence: CD31 (Abcam, ab9498), VE-cadherin/CD144 (Abcam, ab33168), OCT4 (Millipore, MAB4419), NANOG (CST, 4903), and BCL6B (Immunoway, YN0324); (2) flow cytometry: FITC-conjugated anti-CD31 (BD Biosciences, 555445), Alexa Fluor®647-conjugated anti-CD144 (BD Biosciences, 561567), APC-conjugated anti-FLK1 (BioLegend, 359916), PE-conjugated anti-CD73/NT5E (BioLegend, 344003), and APC-Cy7-conjugated CD184/CXCR4 (BioLegend, 306527).

For immunostaining, cells were fixed with 4% paraformaldehyde (PFA) for 10 min. After PBS wash, the fixed cells were permeabilized in PBS containing 0.2% Triton X-100 for 20min. Following blocking in 5% BSA (bovine serum albumin), cells were incubated with primary antibodies at 4℃ overnight. Cells were washed in PBST for 3 times, incubated with fluorophore-conjugated secondary antibodies for 1 hour, washed in PBST, and stained with 1 μg/mL DAPI.

For flow cytometry analysis and sorting, cells were dissociated into single cells using Accutase, washed with PBS. After centrifugation (300×g, 5 min), the cell pellet was resuspended in the FACS washing buffer (PBS with 5% FBS and 2.5 mM EDTA) with fluorophore-conjugated antibodies or IgG, incubated at room temperature for 30 min, washed twice with PBS, and resuspended in FACS washing buffer. Data were collected with BD LSRFortessa flow cytometer

and analyzed using FlowJo Software (version 10.0.7). For Western blot, cells were trypsinized and lysed in RIPA buffer (Applygen) containing 1:50 Protease inhibitor cocktail (Roche). Total proteins were dissolved in loading buffer (CWBIO, CW0027S), denatured at 98℃ for 10 min, separated with 12% SDS-PAGE gel, and transferred to nitrocellulose membrane. The membrane was blocked in TBST containing 5% skim milk and incubated with primary antibodies at 4℃ overnight. Then, the membrane was washed with TBST and incubated with horseradish peroxidase (HRP)-conjugated secondary antibody (EarthOx, E030120). After washing with TBST, the membrane was reacted with chemiluminescent HRP substrate (Merck, WBKLS0100). The protein band was visualized by ECL reaction.

EC functional test assays

The biological function of hPSC-ECs was assessed by human DiI-conjugated acetylated low-density lipoprotein (DiI-ac-LDL) uptake and tube formation assays. For the DiI-ac-LDL uptake assay, ECs were incubated with 5 mg/mL of DiI-Ac-LDL (Yeasen, 20606ES76) at 37 °C for 4 hours, washed with PBS, and stained with Hoechst33258 (Dojindo) to label the nuclei. For the tube formation assay, 1×10^5 ECs were plated onto one well of 24-well plates pre-coated with Matrigel and incubated at 37 °C for 12 hours.

Quantitative PCR

Total RNA was extracted from freshly dissociated cells using TRIzol (Invitrogen) and purified with RNeasy Plus Mini Kit (Qiagen). 1 μg total RNA was reverse transcribed with 5×All-In-One RT MasterMix (Abm). Q-PCR reactions were performed using GoTaq qPCR Master Mix (Promega) in a CFX96 Real-Time thermal cycler (Bio-Rad), and results were analyzed with the Bio-Rad CFX Manager program. The sample input was normalized against the Ct (Critical threshold) value of GAPDH. Primer sequences are listed in **Table S1**.

RNA-seq, ATAC-seq and ChIP-seq library preparation and high-throughput sequencing

RNA-seq library was prepared using the Smart-seq2 protocol (Picelli et al., 2014) with slight modifications. Briefly, 100 ng total RNA was used for reverse transcription and template switching to synthesize cDNA. Then the cDNA was amplified by PCR. After purification, the amplified cDNA was subjected to library construction using the TruePrep^{TM} DNA Library Prep Kit (Vazyme, TD501).

ATAC-seq library was prepared using the adapted Omini-ATAC-seq protocol (Corces et al., 2017). Briefly, 50,000 freshly sorted cells were lysed and centrifuged to pellet the nuclei. The nuclei were transposed by Tn5. Then, the transposed DNA was purified with MinElute PCR Purification Kit (Qiagen) and subjected to library preparation with the TruePrep™ DNA Library Prep Kit.

ChIP-seq was performed using the CUT&Tag protocol (Kaya-Okur et al., 2019), following the instruction of the NovoNGS® CUT&Tag 2.0 kit (Novoprotein, N259-YH01). Briefly, 50,000 freshly sorted cells were immobilized on Concanavalin A magnetic beads and permeabilized. After the primary Ab binding, beads were washed and incubated with the secondary antibody. After washing to remove unbound antibodies, pA/G-Tn5 was added to allow transposition on the DNA regions binding to the Ab. Then, the transposed DNA was released, purified and subjected to library construction using the reagents provided by the kit. Antibodies used in this study for CUT&Tag are anti-H3K4me3 (Merck, 04-745, 1:100), anti-H3K27me3 (Diagenode, C15410069, 1:100), anti-H3K27ac (ActiveMotif, 39133, 1:100), rabbit IgG (CST, 3900, 1:100), guinea pig anti-rabbit IgG (Cloud-clone, SAA544Rb50, 1:100).

All the generated libraries were sequenced on the Illumina X10 platform (paired-end, 150 bp).

ATAC-seq and ChIP-seq data analysis

For ATAC-seq and ChIP-seq data analysis, raw data was first quality controlled (FastQC, version 0.11.7) and trimmed (Trim_Galore, version 0.4.4). The trimmed reads were mapped to the hg38 genome (UCSC) with bowtie2 (version 2.3.5) (Langmead and Salzberg, 2012). Mapped reads were sorted by Samtools (Li et al., 2009), PCR duplicates and reads from mitochondria were removed (Picard tools MarkDuplicates). Then, filtered bam files were transferred to the bigwig file using the bamCoverage tool from deepTools2 (Ramirez et al., 2016). Integrative genomics viewer (IGV) (Robinson et al., 2011) was used to visualize enriched signals. Peaks were called using MACS2 (Zhang et al., 2008; Feng et al., 2012) (version 2.2.6).

For accessible/open chromatin analysis from ATAC-seq data, the identified peak regions were analyzed by Homer (Heinz et al., 2010) *findMotifsGenome.pl* (version 4.10.4) to find enriched TF motifs. For peak intensity analysis, consensus peaks across all samples were first generated. Count and RPKM of the peaks were calculated, differential peaks were extracted with DiffBind (Ross-Innes et al., 2012) and annotated with ChIPseeker toolkits (Yu et al., 2015). Then differential peaks

were classified with K-means clustering. The deepTools2 tool plotMatrix was used for differential peak intensity calculation, and plotHeatmap for data visualization.

For peak annotation and analysis, peaks called from ATAC-seq and ChIP-seq were annotated to their nearest gene according to the following priority order: promoter, 5'-UTR, 3'-UTR, exon, intron, downstream of gene end, and distal intergenic. ClusterProfiler (Yu et al., 2012) was used for peak-associated gene GO (Gene ontology) and KEGG pathway enrichment analysis.

For H3K4me3 broad peak analysis, broad peak was called with MACS2 (*callpeak --broad*), peaks were sorted by peak width, then the top 5% broadest peaks were extracted as signature H3K4me3 markers of each differentiation stage and annotated to their nearest gene.

For CRE (cis-regulatory elements) identification, ATAC-seq peaks sharing at least 20% overlap with H3K27ac and/or H3K4me3 peaks were identified as potential CRE using bedtools (Quinlan and Hall, 2010) (intersect f=0.2). Stage-specific CREs were extracted and annotated to their nearest gene(s).

For TSS region signal intensity analysis, reads distributed ±1kb within gene TSS region were calculated using DiffBind and the RPKM for each region was used for heatmap plotting.

RNA-seq data analysis and identify stage-specific transcription factors

Raw RNA-seq reads were trimmed and mapped against the hg38 reference genome, and then the expression (FPKM and count) was calculated with RSEM (Li and Dewey, 2011) (version 1.2.28). For stage-specific TF identification, RNA-seq data were subjected to (negative binomial tests for) pairwise comparison using the DESeq2 (Love et al., 2014). Stage-specific genes were extracted from the differentially expressed genes list using the following thresholds: FPKM >5 at least in one stage (2 replicates), the sum of FPKM (all samples) > 10, FPKM fold change >2 in one stage over the other stages. All the stage-specific genes were cross-referenced with the gene list downloaded from the AnimalTFDB3.0 database (Hu et al., 2019) to identify the stage-specific TFs of hPSC-EC differentiation. ScRNA-seq data (GSE166462 and GSE131736) was integrated and analyzed using Seurat v4.0 (Hao et al., 2021).

Plasmid construction, lentivirus production and hPSC transfection

The Tet-On system (ClonTech) was used for inducible gene expression. BCL6B coding sequence (CDS) was cloned from EC cDNA and fused with T2A-GFP, then the BCL6B CDS-T2A-GFP cassette was placed behind a TRE promoter-driven lentiviral backbone. The pLenti-rtTA

(Addgene#19780) plasmid was used for constitutive rtTA expression. SgRNAs (**Table S1**) for BCL6B gene mutation were constructed into the pLenti-sgRNA(MS2)-zeo plasmid backbone (Addgene#61427). 293FT cells were used for lentivirus production by Lipofectamine® 2000 (Invitrogen) transfection. The 293FT cells were maintained in DMEM medium supplemented with 10% fetal bovine serum (FBS) and 1mM sodium pyruvate. For gene knock-out using the iCas9 cells (Gonzalez et al., 2014), cells were incubated with sgRNA-expressing lentivirus while passaging, lentivirus was washed away after incubating for 6 hours. Then, 2 μg/mL zeocin (Invitrogen) and 1 μg/mL doxycycline (Sigma) were added to select cells with sgRNA cassette-insertion and to induce Cas9 expression. After selection, single cells were seeded onto a 10-cm dish and grew for 10-14 days to form single colonies. The colonies were picked into a 24-well plate and genotyped. The colonies with BCL6B gene disruption on both alleles were expanded. Similarly, for Tet-on system-mediated BCL6B CDS expression, H1 cells were infected with rtTA and TRE-BCL6B-T2A-GFP lentivirus.

Statistical analysis

Quantitative data are represented as mean ± SEM (standard error of mean). The statistical significance was determined using two-tailed Student's t-test (two-tail) for two groups or one-way ANOVA for multiple groups. A value of $p < 0.05$ was considered statistically significant.

Data availability

Raw and processed RNA-seq, ATAC-seq, and ChIP-seq data are publicly available at the Gene Expression Omnibus (GEO) with accession number **GSE186755**. All other relevant data are available from the corresponding author upon request.

References

Corces, M.R., Trevino, A.E., Hamilton, E.G., Greenside, P.G., Sinnott-Armstrong, N.A., Vesuna, S., Satpathy, A.T., Rubin, A.J., Montine, K.S., Wu, B., *et al.* (2017). An improved ATAC-seq protocol reduces background and enables interrogation of frozen tissues. Nature Methods 14, 959-+.

Feng, J., Liu, T., Qin, B., Zhang, Y., and Liu, X.S. (2012). Identifying ChIP-seq enrichment using MACS. Nat Protoc 7, 1728-1740.

Gonzalez, F., Zhu, Z.R., Shi, Z.D., Lelli, K., Verma, N., Li, Q.V., and Huangfu, D. (2014). An iCRISPR Platform for Rapid, Multiplexable, and Inducible Genome Editing in Human Pluripotent Stem Cells. Cell Stem Cell 15, 215-226.

Hao, Y., Hao, S., Andersen-Nissen, E., Mauck, W.M., 3rd, Zheng, S., Butler, A., Lee, M.J., Wilk, A.J., Darby, C., Zager, M., *et al.* (2021). Integrated analysis of multimodal single-cell data. Cell 184, 3573-3587 e3529.
Heinz, S., Benner, C., Spann, N., Bertolino, E., Lin, Y.C., Laslo, P., Cheng, J.X., Murre, C., Singh, H., and Glass, C.K. (2010). Simple Combinations of Lineage-Determining Transcription Factors Prime cis-Regulatory Elements Required for Macrophage and B Cell Identities. Molecular Cell 38, 576-589.
Hu, H., Miao, Y.R., Jia, L.H., Yu, Q.Y., Zhang, Q., and Guo, A.Y. (2019). AnimalTFDB 3.0: a comprehensive resource for annotation and prediction of animal transcription factors. Nucleic Acids Research 47, D33-D38.
Kaya-Okur, H.S., Wu, S.J., Codomo, C.A., Pledgers, E.S., Bryson, T.D., Henikoff, J.G., Ahmad, K., and Henikoff, S. (2019). CUT&Tag for efficient epigenomic profiling of small samples and single cells. Nature Communications 10.
Langmead, B., and Salzberg, S.L. (2012). Fast gapped-read alignment with Bowtie 2. Nat Methods 9, 357-359.
Li, B., and Dewey, C.N. (2011). RSEM: accurate transcript quantification from RNA-Seq data with or without a reference genome. Bmc Bioinformatics 12.
Li, H., Handsaker, B., Wysoker, A., Fennell, T., Ruan, J., Homer, N., Marth, G., Abecasis, G., Durbin, R., and Genome Project Data Processing, S. (2009). The Sequence Alignment/Map format and SAMtools. Bioinformatics 25, 2078-2079.
Love, M.I., Huber, W., and Anders, S. (2014). Moderated estimation of fold change and dispersion for RNA-seq data with DESeq2. Genome Biol 15, 550.
Picelli, S., Faridani, O.R., Bjorklund, A.K., Winberg, G., Sagasser, S., and Sandberg, R. (2014). Full-length RNA-seq from single cells using Smart-seq2. Nature Protocols 9, 171-181.
Quinlan, A.R., and Hall, I.M. (2010). BEDTools: a flexible suite of utilities for comparing genomic features. Bioinformatics 26, 841-842.
Ramirez, F., Ryan, D.P., Gruning, B., Bhardwaj, V., Kilpert, F., Richter, A.S., Heyne, S., Dundar, F., and Manke, T. (2016). deepTools2: a next generation web server for deep-sequencing data analysis. Nucleic Acids Research 44, W160-W165.
Robinson, J.T., Thorvaldsdottir, H., Winckler, W., Guttman, M., Lander, E.S., Getz, G., and Mesirov, J.P. (2011). Integrative genomics viewer. Nature Biotechnology 29, 24-26.
Ross-Innes, C.S., Stark, R., Teschendorff, A.E., Holmes, K.A., Ali, H.R., Dunning, M.J., Brown, G.D., Gojis, O., Ellis, I.O., Green, A.R., *et al.* (2012). Differential oestrogen receptor binding is associated with clinical outcome in breast cancer. Nature 481, 389-U177.
Yu, G.C., Wang, L.G., Han, Y.Y., and He, Q.Y. (2012). clusterProfiler: an R Package for Comparing Biological Themes Among Gene Clusters. Omics-a Journal of Integrative Biology 16, 284-287.
Yu, G.C., Wang, L.G., and He, Q.Y. (2015). ChIPseeker: an R/Bioconductor package for ChIP peak annotation, comparison and visualization. Bioinformatics 31, 2382-2383.
Zhang, F., Wang, L., Li, Y., Liu, W., Duan, F., Huang, R., Chen, X., Chang, S.C., Du, Y., and Na, J. (2017). Optimizing mesoderm progenitor selection and three-dimensional microniche culture allows highly efficient endothelial differentiation and ischemic tissue repair from human pluripotent stem cells. Stem Cell Res Ther 8, 6.
Zhang, F., Zhu, Y., Chen, J., Kuang, W., Huang, R., Duan, F., Li, Y., Wang, L., Qiu, H., Chen, X., *et al.* (2021). Efficient endothelial and smooth muscle cell differentiation from human pluripotent stem cells through a simplified insulin-free culture system. Biomaterials, 120713.
Zhang, Y., Liu, T., Meyer, C.A., Eeckhoute, J., Johnson, D.S., Bernstein, B.E., Nusbaum, C., Myers, R.M., Brown, M., Li, W., and Liu, X.S. (2008). Model-based analysis of ChIP-Seq (MACS). Genome Biol 9, R137.

Table S1. Oligo sequences used in this study

**Name**	**Accession No.**	**Forward primer**	**Reverse primer**
QP-BCL6B	NM_181844	aggctcaagacacctctggat	cttgtagcggaacgaagaccg
QP-ETV2	NM_014209	ccgacggcgatacctactg	cggtggttagttttggggcat
QP-ACTB	NM_001101	caccattggcaatgagcggttc	aggtctttgcggatgtccacgt
QP-GAPDH	NM_002046	tgatgacatcaagaaggtggtgaag	tccttggaggccatgtgggccat
QP-APLNR	NM_005161	ctctggaccgtgtttcggag	ggtacgtgtaggtagcccaca
QP-NR2F2	NM_001145155	gccatagtcctgttcacctca	aatctcgtcggctggttg
QP-NT5E	NM_001204813	ccagtaccagggcactatctg	tggctcgatcagtccttcca
QP-LYVE1	NM_006691	aggctctttgcgtgcagaa	ggttcgccttttgctcacaa
QP-CXCR4	NM_001008540	actacaccgaggaaatgggct	cccacaatgccagttaagaaga
QP-EFNB2	NM_001372056	aaggactggtactatacccacag	tgtctgcttggtctttatcaacc
QP-NRP1	NM_001024628	gatcctcatcgggcattctctc	actgctctgcaagacactgtag
QP-DLL4	NM_019074	gcccttcaatttcacctggc	caataaccagttctgacccacag
QP-HEY1	NM_001040708	atctgctaagctagaaaaagccg	gtgcgcgtcaaagtaacct
QP-HEY2	NM_012259	aaggcgtcgggatcggataa	agagcgtgtgcgtcaaagtag
QP-NOTCH1	NM_017617	gaggcgtggcagactatgc	cttgtactccgtcagcgtga
QP-NOTCH4	NM_004557	cctggctccttcaactgcc	gcaagtaggtccagacaggt
BCL6B KO gRNA1		actgacgtcacgctgctggt	accagcagcgtgacgtcagt
BCL6B KO gRNA2		cgccactcctccgacgtgct	agcacgtcggaggagtggcg
BCL6B KO gRNA3		ctcaacgagctgcgcctgcg	cgcaggcgcagctcgttgag
BCL6B KO-genotyping		aagaattaggagggaggctgc	ttcggattccctctcaacgg
